# Current Advances in Detection and Treatment of Babesiosis

**DOI:** 10.2174/092986712799828355

**Published:** 2012-04

**Authors:** J Mosqueda, A Olvera-Ramírez, G Aguilar-Tipacamú, GJ Cantó

**Affiliations:** C.A. Salud Animal y Microbiología Ambiental. Facultad de Ciencias Naturales, Universidad Autónoma de Querétaro, Mexico

**Keywords:** Disease, ticks, prevention, cerebral babesiosis, blood smear, reverse line blot hybridization, indirect immunofluorescence, drug target.

## Abstract

Babesiosis is a disease with a world-wide distribution affecting many species of mammals principally cattle and man. The major impact occurs in the cattle industry where bovine babesiosis has had a huge economic effect due to loss of meat and beef production of infected animals and death. Nowadays to those costs there must be added the high cost of tick control, disease detection, prevention and treatment. In almost a century and a quarter since the first report of the disease, the truth is: there is no a safe and efficient vaccine available, there are limited chemotherapeutic choices and few low-cost, reliable and fast detection methods. Detection and treatment of babesiosis are important tools to control babesiosis. Microscopy detection methods are still the cheapest and fastest methods used to identify *Babesia* parasites although their sensitivity and specificity are limited. Newer immunological methods are being developed and they offer faster, more sensitive and more specific options to conventional methods, although the direct immunological diagnoses of parasite antigens in host tissues are still missing. Detection methods based on nucleic acid identification and their amplification are the most sensitive and reliable techniques available today; importantly, most of those methodologies were developed before the genomics and bioinformatics era, which leaves ample room for optimization. For years, babesiosis treatment has been based on the use of very few drugs like imidocarb or diminazene aceturate. Recently, several pharmacological compounds were developed and evaluated, offering new options to control the disease. With the complete sequence of the *Babesia bovis* genome and the *B. bigemina* genome project in progress, the post-genomic era brings a new light on the development of diagnosis methods and new chemotherapy targets. In this review, we will present the current advances in detection and treatment of babesiosis in cattle and other animals, with additional reference to several apicomplexan parasites.

## 1 INTRODUCTION

Babesiosis is a tick-transmitted disease caused by protozoans of the genus *Babesia* and it is characterized by haemolytic anemia and fever, with occasional hemoglobinuria and death[1]. It is a disease with a world-wide distribution affecting many species of mammals with a major impact on cattle and man [2, 3]. With the complete sequence of the *Babesia bovis* genome [4], the post-genomic era brings a new light on the development of diagnosis methods, improved vaccines and new chemotherapy targets. 

Babesiosis was first reported in 1888 by Viktor Babes in Romania who detected the presence of round, intra-erythrocytic bodies in the blood of infected cattle [5]. Babes failed to report the presence of ticks in sick cattle but in 1893, Theobald Smith and Frederick Kilborne of the Bureau of Animal Industry of the United States, published their results of a series of experiments demonstrating that the southern cattle tick *Boophilus *(*Rhipicephalus*)* annulatus* dropping from infected cattle, were responsible for transmitting a disease called the tick fever to susceptible cattle [6]. This observation is considered to be the first to describe a vector arthropod as the carrier of disease. The observations of Smith and Kilborne were strengthened by Cooper Curtice’s own hypothesis that eliminating the cattle tick would eliminate the disease, and they were the basis for the establishment of a tick eradication program, which culminated with the eradication of the southern cattle tick and babesiosis from the United States territory in 1943 [7]. Although there is a quarantine zone in the southern border with Mexico with sporadic outbreaks of ticks and babesiosis [8], it is considered the only successful eradication tick program ever. 

Parasites of the genus *Babesia* infect a wide variety of domestic and wild mammals as well as man [9]. However, the major impact occurs in the cattle industry and the species affecting bovines are the most studied, including *Babesia bovis*, *B. bigemina* and *B. divergens *[2]. Since the times of Babes, Smith and Kilborne, bovine babesiosis has had a huge economic impact due to loss of meat and beef production of infected animals and death. Nowadays to those costs there must be added the high cost of tick control, disease detection, prevention and treatment [10]. Moreover an indirect and underestimated cost of the disease is related with the refusal of cattle farmers in endemic areas to improve the production of beef and milk in their herds by introducing beef or milk-producing, pure-breed animals, most of them from tick-free areas, because they will present an acute form of the disease and many will die in the following weeks to their arrival. The consequence is that the quality of cattle in endemic areas remains low, therefore impeding the development of the cattle industry and the wellbeing of producers and their families [11]. In almost a century and a quarter since the first report of the disease, the truth is: there is no a safe and efficient vaccine available, there are limited chemotherapeutic choices and few low-cost, reliable and fast detection methods. Because of the importance of bovine babesiosis, here we will present the current advances in detection and treatment of babesiosis in cattle and other animals, with additional reference to several apicomplexan parasites. 

## 2 DETECTION METHODS OF BABESIOSIS

The diagnosis of bovine babesiosis is an important tool to control and prevent the dissemination of the disease. During the acute stage of the disease the number of parasites inside the erythrocytes increases in such a way that they can be detected microscopically, however, in chronically infected animals where a subclinical form of the disease occurs, this method is useless and other, more sophisticated methods must be employed. 

### 2.1 Microscopy Detection Methods

Babesia parasites have a complex life cycle as described in Fig. (**1**). Identification of the different stages of the parasite in mammalian or arthropod host tissues can be used for direct diagnosis purposes.

#### 2.1.1 Thin and Thick Blood Smears

**Fig. (2):**
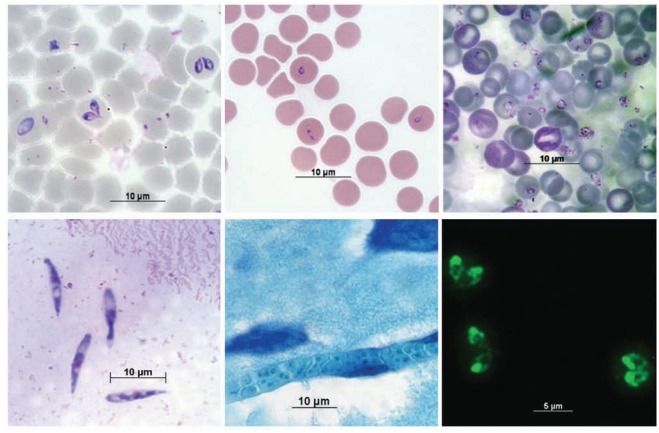
*Babesia* especies in various hosts and tissues. **A**) *Babesia bigemina* in bovine erythrocytes. Blood smear stained with Giemsa. **B**) *Babesia bovis* in bovine erythrocytes. Blood smear stained with Giemsa. **C**) *Babesia microti* in mouse erytrocytes. Blood smear stained with Giemsa. **D**) *Babesia bigemina* kinetes in *Rhipicephalus* (*Boophilus*) microplus haemolymph. Haemolymph smear stained with Giemsa. **E**) *Babesia bovis* in a bovine brain capillar. Histological section of brain tissue stained with Giemsa. **F**) Detection of antibodies against *Babesia bigemina* by the Indirect Fluorescent Antibody Test (IFAT). Bovine antibodies were detected by a secondary, donkey IgG anti- bovine IgG bound to Alexa-Fluor 488. Images were obtained with an objective of 100X.

Vicktor Babes was the first to identify *B. bovis* on thin blood smears of infected cattle [5]. Theobald Smith was also looking for the causative agent of cattle fever for several years but he consistently failed; the reason, he was looking into blood from chronically infected cattle. It was until an acute case came across to him that he could identify the intraerythrocytic corpuscles, in this case *B. bigemina *[12]. Thin blood smears were the first method to detect *Babesia* parasites in clinical samples, a method still used today very effectively in most diagnostic laboratories. Blood is usually collected, combined with an anticoagulant and smeared on a glass slide, air-dried, fixed with methanol, and stained with Giemsa or a similar stain for several minutes. The slide is then washed thoroughly and dried. Intraerythrocytic parasites are observed under a microscope using a 100X objective and a drop of immersion oil. For *B. bigemina,* paired merozoites measure 2.5-3.5 μm of diameter; *B. bovis* merozoites measure 1.5-2 μm of diameter and *B. divergens* merozoites measure 1.5-0.4 μm. They can be detected for up to one infected erythrocyte per ten thousand cells, requiring the analysis of 100-200 fields, the equivalent to 0.5 μl of blood [13, 14]. It is easy to do and inexpensive but requires an experienced microscopist to differentiate species and is reliable only if the amount of parasites in the blood is high enough to be detected, which is usually only possible during acute cases. Special attention must be paid to the source of the blood; peripheral blood is useful only for species like *B. bigemina*, *B. divergens*, or *B. gibsoni,* which do not adhere to the vascular endothelium. Some species like *B. bovis* or *B. canis* adhere to endothelial cells [15, 16] and their diagnosis using this method is feasible if the blood sample is taken directly from a blood capillary from the ear or the skin of the tail, compared with peripheral blood taken from the jugular or caudal veins, since capillary blood contains a higher percentage of infected erythrocytes for these species. The observation of paired intraerythrocytic merozoites is indicative of infection but there are other stages of the parasite like the trophozoites, which present different forms and sizes depending on the species, and these make their detection difficult and time-consuming (Fig. **2A-C**) [17]. 

Another technique developed to detect low levels of parasitemia, especially in cases where *B. bovis* is involved, is based on thick smears of infected blood stained with Giemsa [13, 18]. For this technique, in order to obtain good results, a small drop of blood is placed in the center of the slide and it is fixed by heat, without smearing it, then it is stained routinely, washing it carefully to avoid losing the tissue while the excess of stain is removed. Once it has been dried, it can be observed with a microscope, similarly as the thin smear [19]. The advantage of the thick smear is that a large amount of erythrocytes is analyzed in a reduced amount of space, therefore the probability of finding infected cells is ten times higher than in the thin smear [18]. The method is usually recommended when *B. bovis* infections are suspected or when a subclinical disease occurs. This method relies on a very experienced microscopist who must identify the *Babesia*-infected cells among a mass of conglomerated erythrocytes. 

#### 2.1.2 Brain Smears

When a bovine has died and it is presumed to be from babesiosis caused by *B. bovis* due to presence of nervous clinical signs, identification of the parasite can be done by brain smears. In this case, a small sample of grey matter of the cerebral cortex is placed on a slide and the tissue is smeared using another slide. The brain tissue is fixed and stained as mentioned above. The diagnosis is based upon observation of brain capillaries filled with infected erythrocytes [1]. Almost one hundred percent of erythrocytes present in the brain capillaries are infected (Fig. **2E**) [20]. Smears of other organs as the kidney or liver can also be carried out with good results.

#### 2.1.3 Haemolymph Smears

As a tick-transmitted pathogen, *Babesia* parasites infect several tick tissues. Immediately after repletion with a blood meal, the tick acquires intracellular parasites, which soon escape from the erythrocytes and remain in the gut lumen for a short period of time. Sexual reproduction occurs and infective diploid cells penetrate the midgut cells of the tick and transform to motile stages called kinetes after 72 h post-repletion [21]. Kinetes, are motile stages of the parasite, which reach the haemolymph of the tick and infect several organs including the ovary of mated females. A good approach to identify adult females infected with *Babesia* is detecting kinete stages in the haemolymph by performing the haemolymph test. For this technique, a leg of the tick is cut with small scissors to obtain a drop of haemolymph, which is placed on a glass slide. Usually the drop is too small to be smeared so it is let dry, fixed and stained as a regular blood smear. The diagnosis is based on the observation of the kinetes, which have a vermicular shape and are usually 14.3-16.9 μ long by 2.8-3.4 μ wide, depending on the species (Fig. **2D**) [22, 23]. It is very difficult to differentiate species by the haemolymph test. Based on the biological cycle, kinetes first appear 72 h after engorgement and remain in the haemolymph until the female tick dies, with maximum kinete presence at days 5-6 post repletion [21]. It has been suggested that *Rhipicephalus* (*Boophilus*) ticks have been adapted to start laying their eggs before kinetes reach the ovary and invade the developing embryos; the first eggs are laid at 72 h post repletion and the first infected eggs appear 92 h post repletion. By laying some of the eggs before they are infected by the parasite, female ticks ensure a percentage of their progeny to be *Babesia*-free [24]. The haemolymph test requires an experienced microscopist since female ticks collected from cattle in endemic areas have a very low amount of kinetes. 

### 2.2 Immunological Methods

#### 2.2.1 Indirect Fluorescent Antibody Test (IFAT)

When the number of *Babesia* parasites in the blood is too low to be detected, searching for antibodies against proteins of blood stages has proven to be a reliable tool to identify infected carriers or previously exposed animals. The IFAT was first described by Ristic and Sibinovic in 1964 to detect antibodies against *B. caballi* in chronically infected horses [25]. Since then, it has been adapted to all *Babesia* species and has a good level of specificity and sensitivity [26-31]. The IFAT is based on the recognition of parasite antigens by serum antibodies in the blood of the tested animal. Bound antibodies are detected by a fluorochrome-labelled antibody anti-Ig (secondary antibody). Antigen preparation consist of intraerythrocytic merozoites grown in culture or obtained from infected blood removed of plasma and white cells, so that erythrocytes with a high level of parasitemia (>7%), usually mixed with 5% bovine serum albumin or another soluble protein, are smeared on a glass slide. Once the smears dry, they are wrapped individually and stored at -70°C and in this way they can last for several years. When the antigen is going to be used, the slides are thawed, desiccated and fixed with acetone, which removes the haemoglobin and permeates the cell [32]. IFAT consists of a series of incubation and washing steps, first the serum to be tested is diluted 1:80 and after an incubation period the unbound antibody is removed by washing. A secondary antibody anti-bovine IgG conjugated with a fluorochrome is used to identify bound antibodies. After a second round of washing steps, the smear is dried and analyzed with a fluorescence microscope using specific filters for the fluorochrome. Positive results depend on the observation of fluorescent parasites indicating the presence of anti-*Babesia* antibodies in the tested serum (Fig. **2F**). IFAT is easy to do but requires a good quality antigen, which is difficult to obtain. It also requires an experienced microscopist and a fluorescence microscope. It can discriminate between *Babesia* species although some cross-reaction problems have been reported [33]. Historically, Fluorescein Isothiocyanate (FITC) has been used as the standard fluorochrome for IFAT, however, newer fluorescent dyes like Alexa-488 (Invitrogen, USA) or Dylight-488 (Jackson ImmunoResearch, USA) offer better options due to their improved photostability and brightness, and a wider pH range [11]. 

Detection of anti-*Babesia* antibodies from wildlife species can be achieved by using a dye-conjugated Protein G or A [34]. Originally, Protein G and A are bacterial proteins that bind with high affinity to the Fc region of several classes of antibodies. Commercial, recombinant proteins are available and they are engineered so that the Fab binding domain and the albumin domain present in the native proteins were removed, leaving only their high-affinity Fc domain. For example, antibodies from white-tailed deer (*Odocoileus virginianus*) against *B. bovis* or *B. bigemina* have been detected using Protein G coupled with FITC or Alexa-488 [35, 36]. Since Protein G and A differ in their affinity to bind antibodies from different species, the evaluation and selection of the appropriate protein are necessary.

#### 2.2.2 Enzyme-Linked Immunosorbent Assay (ELISA)

When a large number of serum samples is processed, the IFAT test becomes time-consuming and not very effective; this is mainly because each sample must be analyzed at one time by the diagnostician and reading each sample may take several minutes [37]. Other methods based on automation like ELISA are very useful. ELISA has the advantage of non-subjectivity, capacity to read a large number of samples easily and presents higher specificity than the IFAT. There are several ELISA versions for the detection of several *Babesia *species including *B. bovis*, *B. bigemina*, *B. divergens*, *B. caballi*, *B. canis*, *B. gibsoni*, and *B. microti *[32, 38-40]. This ELISA method was initially performed using purified crude antigen from infected blood, but the cross-reactivity with serum proteins was very high [13]. Recent ELISA methods include the use of recombinant antigens and monoclonal antibodies, thus increasing specificity and diminishing unspecific binding and signal. Modern biotechnology allows the expression of antigenic pathogen proteins, which can be bound on ELISA wells and used to evaluate the presence of anti-babesia antibodies using an anti-IgG conjugated with an enzyme, usually peroxidase [41-44]. A common problem observed in bovine babesiosis diagnosis with this indirect ELISA is the fact that recombinant proteins, especially those expressed in bacteria are often co-purified with bacteria proteins, to which bovine serum reacts strongly, thus giving high background signal, affecting the interpretation and standardization protocols. An approach to overcome this problem is to use a monoclonal antibody against a conserved epitope in the target protein and implement a competitive ELISA (cELISA) format in which unbound antigen is recognized by the monoclonal antibody and this is detected by a labelled Anti mouse-IgG antibody. In this way, the signal observed is the result of the lack of specific bovine antibodies bound to the recombinant protein, thus avoiding noise signal due to unspecific binding. cELISA has been developed for *B. bovis*, *B. bigemina* and *B. caballi* and their use has been validated in several diagnostic laboratories around the world [45-48]. 

#### 2.2.3 Immunochromatography Test (ICT)

The immunochromatographic test is a rapid diagnostic device that detects antibodies against a specific antigen in a small amount of serum by means of specific antibody and a recombinant antigen both impregnated on a nitrocellulose membrane-based test strip [49]. ICT is very convenient because it is very easy to perform and read, therefore does not require a trained technician; it does not use any special equipment, so it can be implemented in the field and it has a low cost comparable with other techniques; it is a fast test taking only ten to fifteen minutes to complete and it is very stable under different temperatures [50]. All of these are very powerful reasons to consider the ICT the immunological diagnostics method of the future, especially for diseases like babesiosis where a rapid, easy to read assay is very much needed under field situations particularly in developing countries where equipment and electricity are limiting. ICT has been developed for *Babesia bovis*, *B. bigemina*, *B. caballi, B. gibsoni* and *B. microti *[51-55]. All of them use rabbit polyclonal antibodies as the base for the test. These tests use recombinant antigens like the merozoite surface antigen 2 (MSA-2) for *B. bovis*; the rhoptry associated protein 1 (RAP-1) for *B. bigemina*; a 48 kDa rhoptry protein for *B. caballi* (Bc48); the P50 antigen of *B. gibsoni *as well as the secreted antigen 1 (BgSA1); and a novel secreted antigen 1 for *B. microti *(BmSA1). The concordances compared with standard methods like ELISA and IFAT have been evaluated for *B. bovis* (92.5 and 90.3%, respectively), *B. bigemina* (96.8 and 92.5%, respectively) and *B. gibsoni* (100 and 85.7%, respectively). Concordance with ELISA has been determined for *B. caballi* (91.8%). Combined ICT designed to detect different species in the same assay has been developed for *B. bigemina*/*B bovis* and for *B. caballi*/*Thelieria equi *[55, 56]. The implementation of tests using highly specific monoclonal antibodies, and those designed to identify the parasites directly from a blood sample, which are very much needed to diagnose acute babesiosis, have yet to be developed; although there is hope since there are already commercial tests for malaria [57]. To date, ICT not yet commercially available in any country. 

### 2.3 Molecular Methods

Immunological methods to detect *Babesia* parasites have the disadvantage of relying on the presence of specific antibodies against those parasites, which may take days or weeks to develop in an infected animal or they are present for months after the infection has disappeared, making their usefulness very limited in acute disease cases, vaccinated animals or cleared-by-treatment animals. Molecular methods aimed to detect nucleic acids have been very useful when immunological methods do not work. Detecting nucleic acids is an indirect way of detecting the parasite so they are still considered indirect methods. However, the sensitivity and specificity of these methods are very high and over the past years many different approaches have been developed to detect *Babesia* species in their hosts and their vectors. 

#### 2.3.1 DNA Probes

Deoxyribonucleic acid probes were the first method developed to detect babesial DNA from parasitized blood. For this, DNA is isolated, purified and cloned while adding a marker, which initially was radioactive. The marked single stranded DNA is then used to hybridize to DNA present in a tissue, or a membrane. The high specificity of DNA alignment allows only the hybridization to complementary DNA, thus recognition of the desired sequence and a very specific diagnosis. Detection is carried out by autoradiography, chemiluminescence or a colorimetric substrate. McLaughlin *et al*., in 1986 developed a cloned *B. bovis* radioactive probe which was used by dot blot hybridization to effectively detect 100 pg of *B. bovis* DNA, estimated to be equal to DNA present in 50 microliters of 1 in 10^5^ infected erythrocytes [58]. Buening *et al*., described a radioactive repetitive DNA probe, which detected 10 pg of *B. bigemina* DNA and as few as 150 infected erythrocytes [59]. Radioactive probes were developed for other species including *B. caballi* [60] but the hazard of radio-labeled material, short life of radioactivity and high maintenance costs made them difficult to establish as standard detection methods. Soon non-radioactive probes were designed using chemi-luminescence or colorimetric approaches [61, 62]. DNA probes have also been used to detect *Babesia* parasites in tick tissues and their high sensitivity was useful for the detection of infected carriers [63, 64]. However, this methodology takes several days to complete, requires a specialized technician and continuous labeling of the probes; all these were disadvantages against newer methods developed afterwards. 

#### 2.3.2 Polymerase Chain Reaction (PCR)

The first descriptions of the polymerase chain reaction to detect *Babesia* were reported in 1992 for *B. bovis* [65], *B. bigemina* [66] and *B. microti* [67]. An adaptation of the technique is the nested polymerase chain reaction (nested PCR), a format in which two pairs of primers are used in two successive PCR amplifications where the second pair is intended to amplify a secondary target within the first amplified product. A nested PCR has been effective for the detection of carrier animals infected with *B. bigemina* and *B. microti* and the sensitivity has been reported to be as low as one and three infected erythrocytes, respectively [66, 67]. When nested PCR has been combined with labelled probes, the sensitivity increases several logs of magnitude. For example, nested PCR for *B. bovis* was reported to detect 1 parasite in 10^6^ erythrocytes, but when the resulted amplified product was detected with a DNA probe, the sensitivity increased to 1 in 10^9^ [68]. Nested PCR is a very sensitive tool but it is more expensive, takes more time and the risk of self-contamination is higher than the one-step PCR protocol. There have been other protocols in which a single-step PCR has been used with good results. For *B. bovis*, a one-step PCR combined with a labelled probe was able to detect 10 parasitized erythrocytes in 500 μl of blood [65]; a similar methodology was developed for *B. caballi *[69]. All these protocols have a high degree of sensitivity and specificity yet, they are time consuming, expensive and require trained personnel, plus the disadvantages of the labelled probes as commented earlier. More recent protocols have been published based on single or nested steps without the use of hybridization steps and with good levels of sensitivity. A one-step PCR was developed for *B. bigemina *with a level of detection of 2 parasites in 10^8^ erythrocytes [70]. *B. gibsoni* was detected in 1.5 μl of blood with a parasitemia of 2 in 10^9^, and *B. ovis* was detected in a blood sample with a parasitemia 1 in 10^8^ [71, 72]. Similar methodologies have been described for *B. caballi* [73] and *B. canis* subspecies [74]. One point worth to mention is that many of the PCR protocols reviewed here were published before the genomics and bioinformatics era, thus primer design, including absence of primer dimmer or loop formation, BLAST analysis, sequence conservation and an appropriate thermocycler protocol were not considered, which means the primers and the protocols could still be optimized, these aspects may be considered when implementing these methodologies in diagnostic laboratories around the world.

#### 2.3.3 Reverse Line Blot Hybridization (RLB)

When multiple genera, species or strains are going to be detected in a sample of blood or tissue, there is a better option than PCR. Reverse line blot hybridization is a technique initially developed for the diagnosis of sickle cell anaemia [75], then it was used to detect multiple serotypes of *Streptoccoci* [76] and three genotypes of *Borrelia* [77]. RLB consists of specific oligonucleotides covalently bound to a membrane by a N-(trifluoracetamidohexyl-cyanoethyl,N,N-diisopropyl phosphoramidite [TFA])-C6 amino linker. The oligonucleotides are applied with a miniblotter in an aligned format. PCR amplified products labelled with biotin, are then hybridized using the miniblotter but in an alignment perpendicular to the oligonucleotides; in this way it is ensured that all the amplified samples are exposed to each specific oligonucleotide. After a series of stringent washes, the membrane is incubated with a streptavidin-peroxidase conjugate and the signal is detected by adding a substrate using chemiluminescence. Visualization of a dot indicates the spot where the amplified PCR product recognized and bound the specific oligonucleotide [78]. This method has been used for the detection of several species of *Babesia* including *B. bovis*, *B. bigemina*, *B. divergens*, *B. major*, *B. motasi*, *B. crassa*, and *B. caballi*, although this technique is mostly used for the combined detection of different genera and species in epidemiological studies [79-83]. The most recognized advantage of RLB is that the membranes can be re-used up to 20 times, thus reducing the overall costs of the procedure [79]. Certainly this technique is valuable when several pathogens, species or strains are present in the same sample. 

#### 2.3.4 Real Time PCR (RT-PCR)

Real Time PCR is a technique that amplifies and quantifies a specific DNA fragment. The DNA amplified is quantified as it is being generated (in “real time”), therefore it determines whether a specific sequence is present in the sample, and it also determines the number of copies of that sequence. RT-PCR does this by detecting a fluorescent signal emitted during the PCR reaction as an indicator of the production of the sequence being generated in each cycle; this is opposite to what happens in during the end-point PCR, where the detection of the product is at the end of the reaction. RT-PCR has many advantages over conventional PCR; it does not require post-PCR analysis because the signal is detected as it is generated, therefore, it is faster and it does not generate expenses due to electrophoretic analysis or photo-documentation. Additionally, the fact that the positive fluorescent signal is detected by the thermocycler, the sensitivity is higher compared with the in-gel analysis of ethidium bromide-stained DNA detected in a conventional PCR, and it has been reported to be at least fourfold [84]. There are several formats for RT-PCR, the most common are based on the use of SYBR Green or TaqMan probes. The first RT-PCR method reported for the quantification of *Babesia* was in 2003 when SYBR Green was used to quantify the transcription of the *Babesia bigemina rap-1* locus genes [85]. Since then several protocols have been published for the quantification of *B. gibsoni*, *B. microti*, *B. bovis*, *B. bigemina*, *B. caballi*, *B. canis*, and *B. orientalis*, [86-93]. The sensitivity of RT-PCR has been reported to be also higher than that of conventional PCR, for example, for *B. bovis *and *B. bigemina*, it was reported to detect 0.75 copies of DNA per μl of blood [87]. Probably the only disadvantage of this methodology is the higher costs of the equipment, which usually doubles or triples the cost of a conventional PCR machine. 

#### 2.3.5 Loop Mediated Isothermal Amplification (LAMP)

LAMP is a detection method that amplifies DNA under isothermal conditions with high efficiency, specificity and speed. LAMP is based on the use of four primers specifically designed to amplify six different sequences on the same target DNA, with the aid of an isothermal DNA polymerase. By using four primers to amplify the same target sequence, the specificity of the amplification in increased, solving in part the background amplification observed in most nucleic acid amplification methods. Because LAMP produces a large amount of DNA, it can be analysed by direct observation of; a) an intercalating fluorochrome like SYBR green, ethidium bromide, etc. [94], b) the turbidity generated by magnesium pyrophosphate precipitation as a result of pyrophosphate ion by-product [95], or c) a newer colorimetric method using the metal ion hydroxy naphthol blue (HNB) as an indicator. This colorimetric assay is reported to be superior to the existing colorimetric assays for LAMP with regard to reducing contamination risks and expenses [96]. Because of this, LAMP does not require of electrophoresis and image documentation post-analysis. Additionally, the isothermal conditions required for the DNA polymerase make the use of a thermocycler dispensable, reducing overall cost and time. The sensitivity of LAMP has been estimated to be of a minimum of six copies of target DNA [94]. *Babesia* species have been detected by LAMP including *B. gibsoni*, *B. caballi*, *B. bovis*, *B. bigemina*, *Babesia canis* and *B. orientalis* [97-101]. It has also identified uncharacterized *Babesia* species infecting sheep and goats in China [102]. For *Babesa bovis* and *B. bigemina* the detection level was increased up to 10^3^ and 10^5^, respectively compared with conventional PCR protocols [99]. Finally, LAMP is a technique with many advantages over other nucleic acid-based detection methods and it may be advantageous in developing countries or laboratories where specialized equipment is absent. 

## 3 ANTI-BABESIA DRUGS

Control of bovine babesiosis can be either by tick management, immunization, anti-babesia drugs or by a combination of these approaches [103]. Chemotherapy of babesiosis is important for controling the disease either to treat field cases or to control artificially induced infections [104]. In the past, treatment of cattle babesiosis was less important than disease eradication, principally in countries were the goal was to eradicate the tick vector; however, chemotherapy has been important to control and prevent babesiosis in some areas of the world [105]. In endemic areas, sick animals should be treated as soon as possible with an anti-parasitic drug. The success of the treatment depends on early diagnosis and the prompt administration of effective drugs [106, 107]. A large number of chemical compounds have been reported to be effective against bovine *Babesia* parasites. Some of them were very specific and effective [105, 106], but many have been withdrawn for several reasons [107]. In addition, supportive therapy such as blood transfusions, anti-inflammatory drugs, tick removal, iron preparations, dextrose, vitamins (B complex), purgatives, and fluid replacements, may be necessary in severe cases of babesiosis [3, 105]. The first specific drug used against bovine babesiosis was trypan blue, which is a very effective compound against *B. bigemina* infections, however, it did not have any effect on *B. bovis* and it had the disadvantage of producing discoloration of animal’s flesh, so it is rarely used [105]. For many years, the babesiacides: quinuronium sulfate, amicarbalide, diminazene aceturate and imidocarb diproprionate were used against bovine babesiosis in most of Europe; however, quinuronium sulfate and amicarbilide were withdrawn because of manufacturing safety issues, and diminazene, which is widely used in the tropics as both a babesiacide and a trypanocide, was withdrawn from Europe for marketing reasons [3, 106]; in addition, the product was also withdrawn from the market in Japan recently and is not approved by the Food and Drug Administration in U.S.A. [108]. 

The indiscriminate use of anti-*Babesia *prophylactic agents, including the administration of the drug at sub lethal blood levels to animals, can produce the development of drug resistant parasites, a problem that will require the development of new drugs [106, 109]. New drugs with a chemotherapeutic effect against babesiosis, with high specificity to the parasites and low toxicity to the hosts are desired to control the disease [110, 111]. Identification of novel drug targets is usually based upon metabolic pathways and cell structure [106]. *Babesia* spp. are Apicomplexa parasites that invade erythrocytes and multiply asexually with a reproductive phase [112], which differ from other Apicomplexa that are able to invade and replicate within nucleated cells. However, *Babesia* is closely related to *Plasmodium *protozoa, which also proliferate within erythrocytes and some drugs can be useful for both of these erythrocyte-invading parasites [106]. In addition, most members of the phylum Apicomplexa harbor a semiautonomous plastid like organelle called apicoplast, which was derived *via *secondary endosymbiotic events from an eukaryotic alga [113]. The apicoplast is essential for long term parasite viability and has been an attractive target for development of parasiticidal drug therapies [114]. In fact, the genome of *B. bovis* has been sequenced and provides a greater understanding of *B. bovis* metabolism and potential avenues for drug therapies [4]. Table **1** summarizes the drugs used for the treatment of bovine babesiosis, their chemical name, doses, administration route and references. Thus, this part of the review will discuss the anti-*Babesia *drugs, novel and in current use. 

### 3.1 Conventional Drugs

#### 3.1.1 Imidocarb

**Fig. (3):**
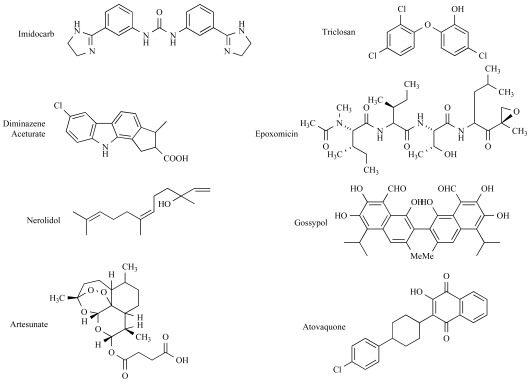
Chemical structures of current and novel drugs against babesiosis.

Imidocarb is a carbanilide derivative [3,3’-bis (2-imidazolin-2-yl)-carbanalidae with antiprotozoal activity (Fig. **3**). It is usually administered either as the dipropionate salt or the dihydrocloride salt. It is the principal babesiacide used in animals, the only one that consistently clears the host of parasites [103, 115] and for over 20 years, it has been used in the treatment and prophylaxis of babesiosis and anaplasmosis [105, 115]. The administration in cattle and sheep, is either by subcutaneous or intramuscular injection [116]. Intravenous injection is not recommended due to its high toxicity, which may cause death in a few minutes [105]. Imidocarb is effective at a recommended dose of 1-3 mg/kg, and it is the drug of choice for bovine babesiosis caused by *B. bigemina,* *B. bovis*, *B. divergens* and *B. caballi* [105]. The mode of action of imidocarb is not fully understood, however, two mechanisms have been proposed: interference with the production or use of polyamines [117], or the prevention of entry of inositol into the parasitized erythrocyte, producing starvation of the protozoan [118]. Imidocarb is associated with residue problems; several studies have showed that imidocarb is retained in edible tissues of ruminants for long periods after treatment [103, 118-121]. Some authors reported high concentrations of imidocarb in the milk of ewes during the first day of treatment, reflecting its fast passage through the blood-milk barrier and a high storage due to the trapping by ionization of the basic drug in milk [122]. The same authors found a low mammary elimination of the drug in goats, probably associated with strong binding to mammary tissue. Therefore, the use of imidocarb in food producing animals has caused some concern. Imidocarb retained in edible tissues has been related with the resistance of the drug to biotransformation processes, as reported in *in vitro* studies on cattle and *in vivo *studies on sheep [119, 123] due to a strong binding of the drug to nuclear components, causing the formation of large deposits principally in hepatocytes, in which imidocarb is accumulated in the cell nucleus [120]. 

#### 3.1.2 Diminazene Aceturate

Diminazene aceturate (4,4´(azoamino) dibenzamidine) is an aromatic diamidine, derived from Surfen (*bis*-2-methyl-4-amino-quinolyl-6-carbamide) (Fig. **3**). It is marketed as a diaceturate salt in a concentration of 45%, in combination with the stabilizer antipyrine (1´2-dihydro-1´5-dimethyl-2-phenyl-3h-pyrazone-3-one), in a concentration of 55%, which is added because of the short stability of the diminazene in water [124, 125]. Diminazene aceturate is the most used anti-trypanosomal agent [126] and it has also been used in the treatment of bovine babesiosis. Diminazene binds irreversibly to double-stranded DNA, in the groove between complementary strands, *via *specific interaction with sites rich in adenine-thymine base pairs [127-129]. The type of binding between diminazene and DNA is non-intercalative and has high affinity for kinetoplastid DNA (kDNA), which impairs kinetoplast replication and function [130]. Moreover, diminazene inhibits the mitochondrial topoisomerase II [128]. Diminazene aceturate is effective against *B. bigemina,* but less effective against *B. bovis* and* B. divergens* [105]. Diminazene aceturate consists of an organic base and organic acid but once dissolved in water, it dissociates. It is usually given by intra-muscular injection at doses of 3-5 mg/kg [105]. It has been observed that the occurrence of relapse, infectious premunity or complete sterile immunity of *Babesia*, has a dose correlation in splenectomised calves [131]. 

### 3.2 Novel Anti-Babesia Drugs 

#### 3.2.1 Triclosan

Triclosan is a chlorinate aromatic compound (2’,4’,4’-tricloro-2’-hydroxyphenil ether), member of a class of synthetic 2-hydroxydiphenylethers, and exhibits broad-spectrum antimicrobial activity [132] (Fig. **3**). It is widely used as a component of deodorant soaps, dermatologic and topical skin preparations, oral rinses, and toothpastes, among others [133]. Triclosan has shown to be effective against ammonia-producing bacteria, yeast, dermatophytes, and certain resident bacteria in the human skin including both Gram-positive and the Gram-negative types. Parasitic protozoa must either acquire lipids from their host or synthesize lipids de novo to produce the new cell membrane during cell replication. Inhibiting the ability to synthesize new membrane prevents the parasite from increasing in surface area, thereby halting cell proliferation and disease progression [134]. Triclosan has demonstrated an inhibitory effect on the growth of *Plasmodium falciparum, Toxoplasma gondii, Theileria parva, Trypanosoma brucei and Perkinsus marinus* in *in vitro studies, *and in *in vivo* studies with *P. berghei *in a mouse model [134-138]. The mode of action of triclosan has been associated with the inhibition of the enoyl-acyl carrier protein (ACP) reductase (FabI), a fatty acid synthase (FAS) located in the apicoplast of *P. falciparum* [136]. FabI reduces the α, β-unsaturated double bonds of the fatty acids bound to the ACP in an NADH or NADPH dependent reaction[139]. NAD+ is the preferred co-factor for the *P. falciparum *FabI enzyme. Triclosan directly binds to FabI, increasing its affinity for the oxidized form of the co-factor NAD+, and thus locking up the enzyme in its NAD-bound form. This leaves no room for NADH binding, which is an essential step, thus bringing FAS and hence the cell growth to a halt [139]. X-ray structural analysis of FabI bound to triclosan demonstrated that the critical binding element of triclosan is the phenol moiety [140]. The fatty acid synthesis pathway is crucial to parasite survival as a result of a key role in membrane construction and energy reduction [141]. *P. falciparum* synthesizes fatty acids with the type II fatty acid system (FAS II), however the type I fatty acid system (FAS I) present in animals and humans is absent; this is in contrast with other apicomplexan parasites such as *Cryptosporidium parvum*, which has no plastid and *T. gondii, *which possesses both fatty acid systems [142]. In relation with *Babesia*, the inhibitory effect of triclosan on the growth of parasites has been reported in *in vitro* culture studies [111]. The growth of *B. bovis *and *B. bigemina *were inhibited at a triclosan concentration of 100 μg/ml, while *B. caballi *and *T. equi *were susceptible to a dose as low as 50 μg/ml. In addition, triclosan prevented parasite re-growth in subsequent subcultures and all stages of the parasite appeared to be affected by the drug. No toxicity against host cells was found following the addition of triclosan [111]. However, the mode of action of triclosan in *Babesia* has not been described yet; the inhibition effect of triclosan is on the fatty acid system but genome-sequencing studies indicated that *Babesia bovis* doesn’t have the fatty acid synthesis pathway in its apicoplast [4], suggesting the presence of a FabI homologue in the chromosomal genome or the existence of another mode of action. Triclosan may also act in *Babesia* upon cellular membranes, perturbing their physical properties and disrupting their function as it has been observed for *T. brucei*, where triclosan acts through other mechanisms not involving the enoyl-ACP reductase [138]. Therefore, additional studies of the mode of drug action and evaluation of the chemotherapeutic effect of triclosan are clearly needed on bovine *Babesia*.

#### 3.2.2 Nerolidol

Nerolidol, also known as peruviol, is a sesquiterpene present in essential oils of several plants (Fig. **3**). It is food-flavoring compound, approved by the U.S. Food and Drug Administration [110, 143]. It has shown an anti-neoplastic activity, and it has been tested as a skin penetration enhancer for the transdermal delivery of therapeutic drugs [143, 144]. Nerolidol has also shown leishmanicidal and anti-malarial activities, due to interference with isoprenoid biosynthesis of the parasites [110, 143, 145]. The apicoplast of apicomplexan parasites has synthesis pathways such as fatty acid biosynthesis and isoprenoid biosynthetic pathways [4]. The synthesis of isopentenyl diphosphate (IPP), the universal isoprenoid precursor, has long been assumed to proceed exclusively *via *the acetate/mevalonate (MVA) ubiquitous pathway, a pathway that is absent from malaria parasites [110, 146]. In *Leishmania amazonensis*, it has been shown that nerolidol inhibits the isoprenoid biosynthesis since it reduces the incorporation of mevalonic acid (MVA) or acetic acid precursors into dolichol, ergosterol and ubiquinone in treated promastigotes due to the blockage of an early step in the mevalonate pathway [143]. In the case of *P. falciparum,* it has been reported that nerolidol interferes with the isoprenoid biosynthetic pathway of the apicoplast, leading to interference with the biosynthesis of dolichols, with the isoprenic chain of ubiquinones, and with protein isoprenylation of the parasites [147]. In the case of *Babesia *parasites, it has been shown *in vitro* that nerolidol can inhibit the growth of *B. bovis, *and *B. bigemina* at 10 μM and* B. ovata, and B. caballi *at 25 μM; the parasite growth is completely suppressed at 50 μM for *B. bigemina*, and at 75 μM for *B. bovis, B. ovata, and B. caballi* [110]. The calculated IC50 values of nerolidol on the third day of culture for the growth of *B. bovis, B. bigemina, B. ovata*, and *B. caballi* were 21±1, 29.6±3, 26.9±2, and 23.1±1 μM, respectively. The mechanism of inhibition of *Babesia* parasites is unknown, nevertheless, it is known that *B. bovis* has an active isoprenoid pathway in its apicoplast, which is similar to that of *P. falciparum* and *T. parva* [4]. Probably *Babesia* parasites have the same mode of action than *P. falciparum.* More studies with neroridol should be done to elucidate its mechanism of action, and its effect in *in vivo* experiments.

#### 3.2.3 Artesunate

Artemisinin and its derivatives such as artesunate, artemether, arteether and dihydroartemisinin are the most common anti-malarial drugs available around the world [108]. They are extracted from a herb called quinghao (*Artemisia annua*—sweet Annie, sweet wormwood), which has been used in the Chinese medicine as an antimalarial herb [148]. Artemisinin is a sesquiterpene triozane lactone, contains an endoperoxide bridge (Fig. **3**) essential for antimalaria activity, and has a broad specificity against *Plasmodium* developmental stages, including activity throughout the asexual blood stages and sexual gametocyte stages [149-151]. Artemisinin has low solubility either in water or oil, and it can only be administered orally. Several semi-synthetic artemisinin derivatives have been developed to improve solubility in both, oil and water, for oral, parenteral and intra-rectal routes [149]. These derivatives are the most important classes of anti-malarial drugs and have a strong impact on modern malaria treatment regimens [152]. Artesunate is a water soluble half-ester succinate derivative, it is the most common derivative and it is very effective in the treatment of severe malaria cases [150]. The major mode of action of artemisinins is by inhibiting a mammalian Ca^2+^ transporting ATPase (SERCA-sarcoplamic/endoplasmic reticulum Ca^2+^ ATPase). SERCA’s role is to reduce cystolic free calcium by actively concentrating Ca^2+^ into membrane bounds vacuoles, an activity critical to cell survival. Then, artemisinins act by inhibiting PfATP6 (a SERCA orthologue enzyme) outside the food vacuole after activation by iron in *P. falciparum *[150]. Moreover it has been showed that artemisinins targets are the calcium dependent ATPases in *Toxoplasma gondii *[153]*. *However other studies showed that the SERCA inhibition of artemisinins is not clear and it is not the only mode of action of artemisinins [150]. Artemisinins can accumulate within neutral lipids and cause parasite membrane damage [154]. Also, over-expression of enzymes associated with oxidative stress, such as catalase, superoxide dismutase II, thioredoxin reductase, γ-glutamylcysteine synthase and several members of the glutathione-S-transferase (GST) family, have shown to reduce susceptibility of tumor cells to artemisinins [155-157]. Artesunate has shown a growth inhibitory effect on *B. caballi, *with doses of 1.0 μg/ml, in *in vitro *cultures. However, artesunate is able to destroy *Theileria equi *but unable to destroy *B. caballi* [158]. Artesunate inhibits the growth of* B. bovis* and *B. gibsoni,* at concentrations equal to or greater than 2.6 μM by day 3 post-treatment in a dose-dependent manner. Artesunate was also effective in the treatment of mice infected with *B. microti* at doses equal to, or greater than 10 mg/kg of body weight on days 8–10 post-infection without side effects, suggesting that artesunate could be a potential drug for *Babesia* infection [108]. 

#### 3.2.4 Epoxomicin

Epoxomicin belongs to a family of α´, β´-epoxyketone natural products characterized by a linear peptide structure [159] (Fig. **3**). Epoxomicin was initially isolated from an *Actinomycetes *strain and it is a potent proteasome inhibitor [160]. The proteasome, a prototypic T1 threonine-peptidase multi-subunit complex plays a key role in endogenous protein turnover. Epoxomicin covalently binds the LMP7, X, Z and MECL1 catalytic β subunits of the proteasome, resulting in inhibition of three of its known proteolytic activities, primarily the chymotrypsin-like activity although, the trypsin-like activity and the peptidyl-glutamyl peptide hydrolyzing (PGPH) activity also can be inhibited [160]. These inhibitory effects cause cell death by promoting the accumulation of ubiquitinated proteins within the cytoplasm [160, 161]. Proteasome inhibitors like epoxomicin have been proposed as anti-protozoa drugs. In the case of apicomplexan parasites, epoxomicin inhibits the growth of the organisms because it blocks the catalytically active proteasomal subunits[162]. For example, the inhibitory effect of epoxomicin has been shown in *Plasmodium falciparum,* where parasites throughout the asexual, sexual, and mosquito midgut stages were effectively killed [163, 164]. Epoxomicin has shown inhibitory effects on the *in vitro *growth of bovine and equine *Babesia* parasites and *in vivo *growth of *Babesia microti *[161]. *Babesia bovis *was significantly inhibited by 10 nM epoxomicin, while a 5 nM epoxomicin treatment inhibited the growth *of Babesia bigemina, Babesia ovata, Babesia caballi, and Theileria equi.* Moreover, in the presence of 50 nM epoxomicin, the growth of *B. bigemina *and *T. equi* was completely suppressed. An epoxomicin concentration of 100 nM was needed to completely suppress the growth of *B. bovis, B. ovata, *and *B. caballi*. Furthermore, combinations of epoxomicin with diminazene aceturate potentiated its inhibitory effect in *in vitro *cell cultures. Additional studies are required to corroborate whether epoxomicin acts in *Babesia* species inhibiting the proteasome activity as in malaria parasites. An interesting observation is that epoxomicin effectively inhibits NF-κβ-mediated proinflammatory signaling and inhibits *in vivo* inflammation [160]. Some *Babesia* species like *B bovis* or *B. canis* cause disease characterized by a systemic inflammatory response [165, 166]. The effect of epoxomicin on this response must also be evaluated since a babesiacidal drug with anti-inflammatory effects should be desirable for the treatment of the disease caused by these species.

#### 3.2.5 Gossypol

Gossypol is a polyphenolic yellow pigment (1,1’,6,6’,7,7’-hexahydroxy-5,5’-diisopropyl-3,3‘dimethyl [2,2’-binaphthalene] 8,8‘-dicar-boxaldehyde) (Fig. **3**), found naturally in pigment glands of the roots, leaves, stems, and seeds of the cotton plant genus *Gossypiurn* [167, 168]. It is a natural toxin that protects the plant from insect damage. Species and varieties of cotton plants differ in concentrations of gossypol present in the seed [169]. Gossypol has been described as an inhibitor of the l-lactate dehydrogenase in *Plasmodium falciparum *(pfLHD) [170]. The pfLDH is essential for the anaerobic phase of parasite growth and it provides energy to the parasite by catalyzing the lactate from piruvate, which is the end product of glucose degradation, using nicotinamide adenine dinucleotide (NAD+) or its reduced form (NADH) as a cofactor [171, 172]. The l-lactate is considered as a prominent product of the transition phase from aerobic to anaerobic metabolism and it is known that in *P. falciparum*, pfLHD plays important roles in regulating glycolysis (Embden–Meyerhof pathway) and balancing the parasitic reduced/oxidized state [172]. The most advanced targets of antimalarial structure-based drug design is pfLDH, and the researchers has been focused in finding pfLDH inhibitors, such as gossypol derivatives [170]. Furthermore, the LDHs of *Toxoplasma gondii *(tgLDH1 and tgLDH2) have been suggested as drug targets for enzyme inhibitors such as gossypol [173]. In the case of *Babesia* parasites, a novel *Babesia bovis *cDNA clone was characterized and genetic analysis showed that it contained an open reading frame of 993 bp, which was considered to encode a *B. bovis *l-lactate dehydrogenase (BbLHD), having high amino acid identities to the LDHs of other protozoans such as *Plasmodium falciparum *and *Toxoplasma gondii *[174]. Also, native BbLDH was expressed in the cytoplasm and the membrane of infected erythrocytes, suggesting the possibility that the BbLDH might spontaneously escape the parasitic body and partially imitate the function of the host LDH, which is essential for the anaerobic glycolysis pathway of the host red blood cells, exchanging to a suitable environment for parasitic survival. However, the exact mode of action of BbLDH has not been elucidated. The inhibition activity of gossypol on the growth of *in vitro Babesia bovis* cultures was 100µM, which represents an IC50 value of 50 μM [174]. This suggests that gossypol can be a babesiadae drug, however, non-ruminant and pre-ruminant animals are particularly sensitive to the toxic effects of gossypol, and in many non-ruminant species, it causes infertility in males as well [169]. In pregnant cows, no effects of gossypol on a superovulation response or embryo development were observed [175]. Therefore more research should be done on the efficacy of gossypol and its derivatives against *Babesia* as well as on the effects in the vertebrate host.

#### 3.2.6 Atovaquone

Atovaquone (1,4-hydroxynaphthoquinone) (Fig. **3**) is an anti-protozoal compound that has broad-spectrum activity against apicomplexan parasites [176] such as *Plasmodium *spp., *Pneumocystis carinii*, *Toxoplasma gondii *and *Babesia *spp. [177, 178]. The mode of action of atovaquone against protozoa parasites is by inhibiting the rate of oxygen consumption [179]. In most eukaryotes the electron transport chain is central to the energy metabolism of metabolically active cells. In higher organisms the electron transport chain is composed by four membrane-enzyme complexes in the mitochondrial inner membrane: NADH-ubiquinone oxidoreductase (Complex I), succinate: ubiquinone oxidoreductase (complex II), ubiquinol:cytochrome c oxidoreductase (Complex III or cytochrome bc1), and cytochrome c oxidase (complex IV or cytochrome aa3), with ubiquinone (coenzyme Q) and cytochrome c functioning as electron carriers between the complexes [180]. In many apicomplexan parasites complex I of the electron transport. However, they have the activity of complex II, III and IV, but reducing equivalents from NADH can be donated directly to ubiquinone in the mithocondrial membrane by a single-subunit NADH dehydrogenase [181]. Atovaquone acts as an active protozoal ubiquinone analogue, which is a mitochondrial protein involved in the electron transport chain [177]. Ubiquinone (also called coenzyme Q) is an integral component of electron transport system in aerobic respiration. Ubiquinone transfers electrons from the primary substrates to the oxidase system at the same time that it transfers protons to the outside of the mitochondrial membrane. Ubiquinone accepts electrons from dehydrogenase enzymes and passes them to electron transport cytochromes. The passage of electrons from ubiquinone to cytochrome *bc*1 (complex III) requires binding of coenzyme Q complex III at the Qo cytochrome domain [177]. Atovaquone binds the Qo cytochrome domain (Complex III) and the site of inhibition locates between cytochromes *b* and *c*_1_ of this complex with an irreversibly bound to a polypeptide with an approximate molecular mass of 11,500 Da [182]. Atovaquone inhibited the respiratory chain of rodent malaria parasites and caused the collapse of their mitochondrial membrane potential in *in vivo *assays [183]. 

Several parasite enzymes linked to the mitochondrial electron transport system are inhibited, such as dihydroorotate dehydrogenase (DHOD), which catalyzes the oxidation of dihydroorotate to orotate and is required for the biosynthesis of pyrimidines. Because plasmodia parasites are unable to scavenge pyrimidines for DNA synthesis and they are required to be synthesized *de novo*, inhibition of DHOD results in parasite death [177, 184]. In *Babesia bovis*, the mitochondrion also helps to synthesize the pyrimidine nucleotides *de novo*, through the passage of electrons by DHOD, which is linked to a respiratory chain *via *ubiquinones [185]. Atovaquone seems to be very efficient against *B. divergens* infecting human erythrocytes *in vitro *[106]. Atovaquone plus azithromycin have demonstrated superiority compared to clindamycin and quinine in the prevention and treatment of experimental babesiosis in hamsters [186, 187]. Also, an antibiotic regimen based on a combination of atovaquone and azithromycin in humans is generally superior to a combination of clindamycin and quinine for the treatment of babesiosis principally in immunocompetent adult patients and in others that do not have tolerance for clindamycin and quinine [188]. Atovaquone has been proved to be effective against *B. divergens* in gerbils (*Meriones unguiculatus*) [189]. Acute fulminating infections were effectively treated with as little as 1.0 mg/kg with increasing effectiveness up to 10 mg/kg. Atovaquone has shown to be a new drug to effectively inhibit the growth of* Babesia* divergens, although the effect on other *Babesia* species should be investigated as well as the mode of action in the mitochondria.

#### 3.2.7 New Drugs Under Research 

Recently, the identification of new drug targets for the control of babesiosis has been described [190]. Cysteine proteases (CP) are therapeutic targets of inhibitors of some apicomplexan parasites [191, 192]. In *Babesia*, it that been reported that cysteine proteases reduce *in vitro* invasion of erythrocytes and the growth of *B. bovis *[191]. A gene belonging to the C1 family of CP from *B. bigemina* (called babesipain-1) was previously identified. A series of new artemisinin-dipeptydil vinyl sulfone hybrid molecules were found as inhibitors of babesipain-1, being effective on the range of 0.3–30 μM, depending on the core-containing molecule [190]. Furthermore, three cathepsin L-like cysteine proteases (BbiCPL 1–3), were identified in the *B. bigemina* genome [193]. Recently, BbiCPL1 was cloned and expressed as a fusion protein and a recombinant BbiCPL1 was obtained, which has activity against typical peptide substrates of cysteine proteases [194]. This finding will allow researchers to screen for specific inhibitors against babesiosis in large-scale studies. BbiCPL1 and the *B. bovis* ortholog (named bovipain-2) were immuno-localized to an undefined intracellular organelle and to the cytoplasm of infected erythrocytes, suggesting that BbiCPL1 may have its function in host cytosol environment or in an intracellular organelle [195]. More research should be done on the molecular mechanisms of these CP on *Babesia* spp., as well as other, yet undiscovered drug targets for the treatment of babesiosis.

## 4 CONCLUSIONS

Detection and treatment of babesiosis are important tools to control babesiosis. Microscopy detection methods are still the cheapest and fastest methods used to identify *Babesia* parasites although their sensitivity and specificity are limited. Newer immunological methods are being developed and they offer faster, more sensitive and more specific options to conventional methods, although the direct immunological diagnoses of parasite antigens in host tissues are still missing. Detection methods based on nucleic acid identification and their amplification are the most sensitive and reliable techniques available today; they are fast, very specific and although most of them relay on sophisticated equipment, new methodologies are being developed without the need of expensive apparatus. Importantly, most of those methodologies were developed before the genomics and bioinformatics era, which leaves ample room for optimization. For years, babesiosis treatment has been based on the use of very few drugs like imidocarb or diminazene aceturate. Recently, several pharmacological compounds were developed and evaluated, offering new options to control the disease. With the complete sequence of the *Babesia bovis* genome and the *B. bigemina* genome project in progress [4], the post-genomic era brings a new light on the development of diagnosis methods and new chemotherapy targets.

## Figures and Tables

**Fig. (1):**
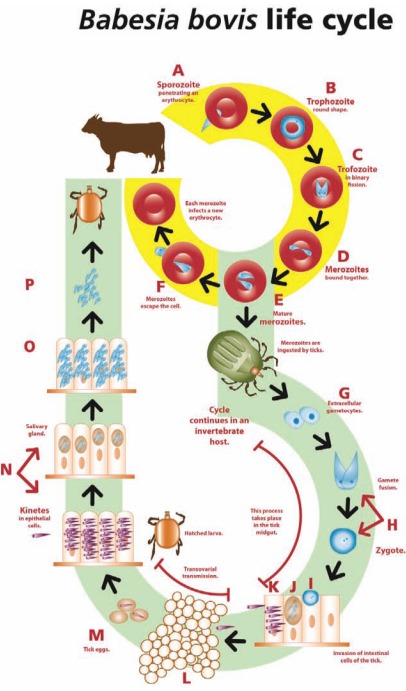
The life cycle of *Babesia bovis*. **A**. A *B. bovis* sporozoite invades an erythrocyte and transforms into a trofozoite. **B**. The trofozoite in a ring shape. **C**. Two merozoites are generated from each trofozoite by binary fission. **D**. Merozoites are initially bound together resembling two pears in an acute angle. **E**. The mature merozoites separate before escaping the erythrocyte. **F**. Merozoites are liberated from the erythrocyte. Some of them will invade new erythrocytes and develop into trofozoites, while others will be picked up by adult ticks to continue their cycle in the invertebrate host. **G**. Sexual stages are freed from the red blood cells in the intestinal tick lumen and develop to gametocytes. **H**. The gametocytes transform into male and female gametes that form a zygote after fusion. **I**. The zygote develops into an infecting stage and penetrates the tick intestinal cells. **J**. Fission bodies form and from them motile kinetes develop. **K**. Kinetes destroy the intestinal cells, escape into the haemolymph and distribute into the different cell types and tissues, including the ovaries. **L**. In the ovary, embryo cells are infected by kinetes (transovarial transmission). **M**. When the female tick lays her eggs, the embryos are already infected. **N**. Hatched infected larvae attach to a bovine and the kinetes migrate to the salivary glands of the tick, where they form a sporoblast. **O**. Thousands of sporozoites develop from each sporoblast. **P**. Tick larvae feed from the bovine blood and the sporozoites are liberated with saliva into the animal’s circulatory system.

**Table 1.** Chemical Drugs Used to Treat Babesiosis

Compound	Chemical name	* Babesia* spp.	Dose	Rout	Current use	References

**Imidocarb**	3,3’-bis (2-imidazolin-2-yl)-carbanalidae	*B. bovis*	1-3 mg kg^-1^	IM, SC	Yes	[105]
		*B. bigemina*				
		*B. divergens*				
		*B. caballi*				

** Diminazene aceturate**	4,4´(azoamino)dibenzamidine	*B. bovis*	3-5 mg kg^-1^	IM	Yes	[105]
		*B. bigemina*				
		*B. divergens*				
		*B. caballi*				

**Nerolidol**	*cis*-3,7,11-Trimethyl- 1,6,10-dodecatrien-3-ol	*B. bovis*	10µM	-	On research	[110]
		*B. bigemina*	25 µM			
		*B. ovata*				
		*B. caballi*				

** Artesunate**	3R,5aS,6R,8aS,9R,10S,12R,12aR)-decahydrido-3,6,9-trymetyl-3,12-epoxy-12H-pyranol(4,4/l-1,2-benzodioepin-10-ol hydrogen succinate	*B. bovis*	2.6 µM	-	On research	[108]
		*B. gibsoni *	10 µg ml^-1^			
		*B. caballi*	10 mg kg^-1^			
		*B. microti*				

**Triclosan**	2’,4’,4’-tricloro-2’-hydroxyphenil ether	*B. bovis*	100µg ml^-1^	-	On research	[111]
		*B. bigemina*	50 µg ml^-1^			
		*B. caballi*				

** Epoxomicin **	α´,β´-epoxyketone	*B. bovis*	10 nM	-	On research	[161]
		*B. bigemina*	5 nM			
		*B. ovata*	0.05-0.5 mg kg^-1^			
		*B. caballi*				
		*B. microti*				

**Gossypol**	1,1’,6,6’,7,7’-hexahydroxy-5,5’-diisopropyl-3,3‘dimethyl [2,2’-binaphthaleneI8,8‘-dicar-boxaldehyde.	*B. bovis*	100µM	-	On research	[174]

** Atovaquone**	1,4-hydroxynaphthoquinone	*B. divergens*	1 mg kg^-1^	-	On research	[189]

IM Intramuscular, SC Subcutaneous.
